# Population turnover, behavioral conservatism, and rates of cultural evolution

**DOI:** 10.1093/beheco/arae003

**Published:** 2024-01-17

**Authors:** Mark Dyble, Alberto J C Micheletti

**Affiliations:** Department of Archaeology, University of Cambridge, Henry Wellcome Building, Fitzwilliam Street, CB2 1QH Cambridge, UK; Department of Anthropology, University College London, 14 Taviton Street, WC1H 0BW London, UK

**Keywords:** behavioral conservatism, culture, population turnover, social learning

## Abstract

Cultural evolution facilitates behavioral adaptation in many species. The pace of cultural evolution can be accelerated by population turnover, where newcomers (immigrants or juvenile recruits) introduce adaptive cultural traits into their new group. However, where newcomers are naïve to the challenges of their new group, population turnover could potentially slow the rate of cultural evolution. Here, we model cultural evolution with population turnover and show that even if turnover results in the replacement of experienced individuals with naïve ones, turnover can still accelerate cultural evolution if (1) the rate of social learning is more than twice as fast as the turnover rate and (b) newcomers are more likely to learn socially than behaviorally conservative existing group members. Although population turnover is a relatively simple factor, it is common to all animal societies, and variation in the turnover rate may potentially play an important role in explaining variation in the occurrence and rates of adaptive cultural evolution across species.

## Teaser

Many animal species possess culture. Since culture relies on social transmission, rates of cultural evolution are determined not only by cognition but also by demography. Here, we use a mathematical model to show that even if population turnover replaces experienced group members with juveniles or migrants not previously exposed to the cultural traits of a group, this turnover can accelerate cultural evolution if incoming individuals are more open to social learning than existing group members.

## INTRODUCTION

Social learning is a key driver of adaptive behavioral plasticity, and many species possess cultural traditions that evolve through time (Whiten et al. 1999; Rendell and Whitehead 2001; Rutz et al. 2018; Aplin 2019; Whiten 2021; Garland et al. 2022; Williams and Lachlan 2022). For humans, cultural evolution is critical to our success, allowing us to adapt to life in a wide range of environments (Foley and Lahr 2011; Gamble 2014; Migliano and Vinicius 2022). What determines the pace of cultural evolution? As well as a range of social, environmental, and cognitive factors (Gruber et al. 2022), one important driver is thought to be dispersal. This is because dispersal (in the sense of individuals leaving one social group and joining another, rather than spatial movement in itself) can result in the transmission of adaptive cultural traits between groups (Powell et al. 2009; Dyble 2018; Mesoudi 2018). In humans, it has been argued that the high levels of mobility between residential groups within hunter–gatherer societies promote rates of cultural evolution far greater than would be expected, given the relatively small size of residential bands (Hill et al. 2014; Migliano et al. 2020). Similarly, theoretical modeling suggests that increased migration between populations could explain bursts of cultural complexity in the archaeological record (Shennan 2001; Henrich and Heinrich 2004; Powell et al. 2009; Creanza et al. 2017).

An assumption made in these models of migration and cultural evolution is that cultural traits from one population will be adaptive in another such that newcomers may bring adaptive cultural traits with them when they move (Powell et al. 2009; Creanza et al. 2017). While this may often be the case, it is not necessarily so, and it is unclear what the consequences would be when dispersing individuals do not possess traits that are adaptive in the group they are joining. An extension of this is to consider the effect of demographic turnover on rates of cultural evolution: what happens when experienced older individuals die and are replaced by inexperienced juveniles? One prediction might be that this population turnover would slow down the rate of cultural evolution since it involves the replacement of experienced individuals with naïve ones. However, an alternative possibility is raised by a recent experimental study on great tits (*Parus major)* by Chimento et al. (2021). In their experiment, semi-wild great tits were given a foraging puzzle with two solutions, one more efficient than the other. The inefficient solution was taught to the birds present at the start of the experiment. The efficient solution was sometimes spontaneously adopted and could then spread through a group via social learning. In one experimental condition, birds were kept in a group of fixed membership. In the other condition, experimenters regularly replaced established birds with naïve wild birds that had never encountered the foraging puzzle before. In both conditions, all birds were able to observe and learn socially from other group members. Although the “population turnover” condition involved the replacement of experienced birds with naïve ones, the efficient solution tended to spread faster in these groups than it did in the groups of fixed membership. This result appears to be a consequence of behavioral conservatism among established birds who continued to repeat the inefficient solution even after groupmates had adopted the efficient one. The consequence of this behavioral conservatism was that immigrant individuals were more likely to adopt the efficient solution, such that population turnover accelerated the spread of the efficient solution. Although this experiment involved population turnover through migration, it raises the possibility that demographic turnover (where experienced but behaviorally conservative individuals die and naïve juveniles replace them) could similarly increase rates of cultural evolution.

To understand the possible influence of population turnover on rates of cultural evolution more generally, we developed a mathematical model of population turnover and social learning. While the model is inspired by the results of Chimento et al., it is not intended to replicate the dynamics of that study exactly. Rather, we seek to identify conditions under which the introduction of naïve individuals into a group can accelerate cultural evolution. Our model is general enough to apply to both population turnover through migration and population turnover through a demographic change where established individuals die, and young individuals (“recruits”) are born.

## METHODS

**Figure 1:**
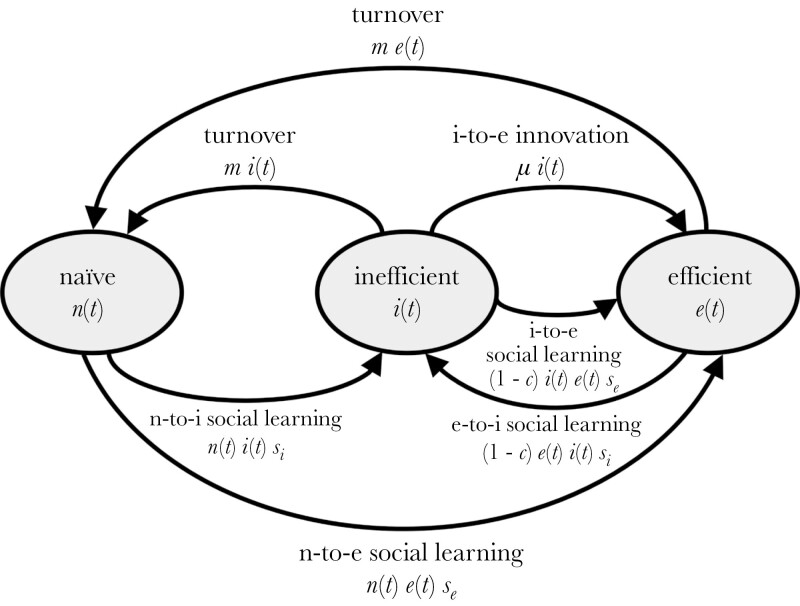
Flow diagram of the model. Arrows represent flows between states due to innovation, turnover, and social learning. Expressions show the extent of these flows.


We developed a compartmental model (Otto and Day 2007) of population turnover and social learning. The model considers a group of individuals facing a problem with two solutions: an efficient solution and an inefficient solution. A fraction of the group *n*(*t*) are naïve and know neither solution, a fraction *i*(*t*) use the inefficient solution, and a fraction *e*(*t*) use the efficient solution. Population size remains constant; *n*(*t*) + *i*(*t*) + *e*(*t*) = 1. The model starts with a population of inefficient individuals. Within each timestep, three processes occur: innovation, turnover, and social learning (Figure 1). We adopt a continuous-time approach so as to harness the power of differential equations, and therefore, we do not assume that these processes occur in any particular order (Otto and Day 2007). Innovation involves inefficient individuals independently discovering and adopting the efficient solution with rate *μ*. Turnover involves individuals from all compartments being substituted by naïve individuals at a rate *m*. In social learning, individuals first encounter individuals from another compartment at a rate equal to the product of their frequencies: a common mass-action assumption. Following an encounter, we assume that naïve individuals always attempt to learn socially but that individuals who already use the efficient or inefficient solutions show a degree of behavioral conservatism, attempting to learn from the individual they encounter with probability 1 – *c* (where 0 ≤ *c* ≤ 1 is “conservatism”). Attempts to learn from inefficient individuals are successful at a rate *s*_i_ and attempts to learn from efficient individuals are successful at a rate *s*_e_. We assume that the efficient solution is more likely to be copied once encountered, capturing this by assuming that *s*_e_ > *s*_i_. Encounters with naïve individuals do not lead to social learning. Changes through time in the relative sizes of the naïve, inefficient, and efficient compartments are described by this system of differential equations:

$$\begin{array}{l} n^{\prime }\left(t\right)=mi\left(t\right)+me\left(t\right)-n\left(t\right)e\left(t\right)s_{e}-n\left(t\right)i\left(t\right)s_{i} \end{array}$$ (1)

$$\begin{array}{l} i^{\prime }\left(t\right)=-\mu i\left(t\right)-mi\left(t\right)+n\left(t\right)i\left(t\right)s_{i}+\left(1-c\right)e\left(t\right)i\left(t\right)s_{i}-\left(1-c\right)i\left(t\right)e\left(t\right)s_{e} \end{array}$$ (2)

$$\begin{array}{l} e^{\prime }\left(t\right)=\mu i\left(t\right)-me\left(t\right)+n\left(t\right)e\left(t\right)s_{e}-\left(1-c\right)e\left(t\right)i\left(t\right)s_{i}+\left(1-c\right)i\left(t\right)e\left(t\right)s_{e} \end{array}$$ (3)

As this system cannot be solved analytically, we study how the relative proportions of naïve, inefficient, and efficient individuals change over time using numerical methods. We investigate the equilibrium values for the three compartments analytically (see Supplementary Material). Note that we assume no “cultural loss”: individuals with the efficient solution never spontaneously revert to the inefficient solution (they may only do so through social learning). As a result, the inefficient solution eventually goes extinct in all cases. Although we start with a population of inefficient individuals, the equilibrium value for the efficient solution (e*) is robust to starting conditions with the exception of a population initially composed of entirety naïve individuals (see Supplementary Material).

## RESULTS

**Figure 2:**
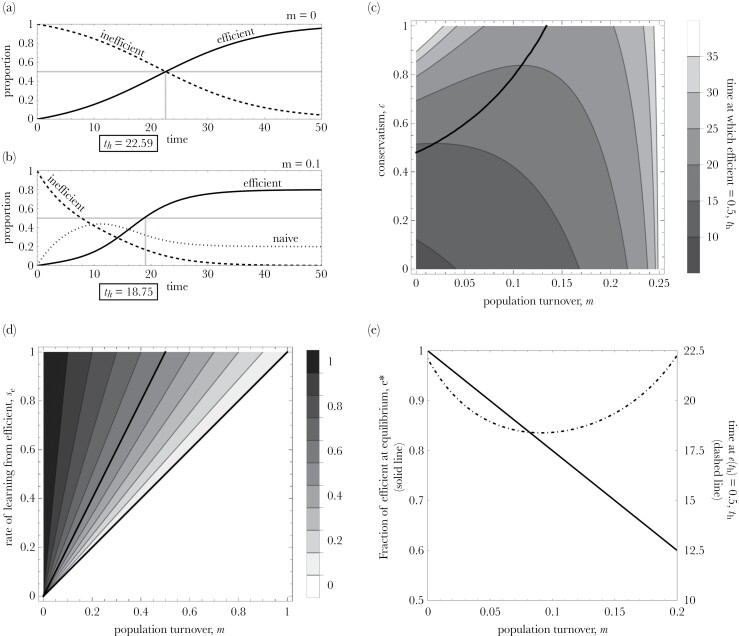
Main results of the model. (a,b) Changes in time in the proportions of efficient (e(t), solid line), inefficient (i(t), dashed line), and naïve (n(t), dotted line) individuals with turnover *m* = 0 and *m* = 0.10 where *s*_e_ = 0.5 and *c* = 0.75. (c) Time *t*_h_ at which half the population possesses the efficient solution, that is, *e*(*t*_h_) = 0.5 (lighter shades for increasing values of time) as a function of m and c. For values on the black line, turnover neither accelerates nor slows cultural evolution; *s*_e_ = 0.5. (d) Fraction of efficient individuals at equilibrium, e^*^, as a function of m and s_e_. (e) Fraction of efficient individuals at equilibrium (e^*^, solid line), and time at *e*(*t*_h_) = 0.5 (*t*_h_, dashed line) as a function of *m*; *s*_e_ = 0.5, *c* = 0.75. The starting points are *e*_0_ = 0, *i*_0_ = 1, *n*_0_ = 0; μ = 0.01, *s*_i_ = 0.1.

We find conditions under which increasing population turnover will accelerate cultural evolution even if newcomers do not possess adaptive cultural traits. For example, in Figure 2a,b, we see that increasing turnover from *m* = 0 (panel *a*) to *m* = 0.1 (panel *b*) reduces *t*_h_, the time taken for the efficient solution to spread to half of the population (*e*(*t*_h_) = 0.5). This occurs not because newcomers possess relevant cultural knowledge (they arrive naïve) but because they are more likely to learn socially than established group members who are behaviorally conservative (in panels *a* and *b*, newcomers always attempt to learn socially, while established individuals attempt to learn socially with probability 0.25).

To generalize this result, we explored values of *t*_h_ (the time at which half the population possesses the efficient solution, *e*(*t*_h_) = 0.5) across a range of values for population turnover, *m*, and behavioral conservatism, *c.* Using numerical methods, we obtain a line where *t*_h_ does not vary with *m* (see Supplementary Material for details). In the region above this line, increasing turnover accelerates cultural evolution (Figure 2c). Below this line, increasing turnover slows cultural evolution. Though the exact values of *t*_h_ depend on parameter values, altering the rates at which efficient and inefficient individuals are copied (*s*_*e*_ and *s*_*i*_, respectively) does not change our results qualitatively, as long as efficient individuals attract more learners (*s*_e_ > *s*_i_; Supplementary Figure S1). Increasing *s*_e_ enlarges the parameter space under which increased turnover accelerates cultural evolution because naïve individuals switch more readily to the efficient solution. Although the parameter space within which increased turnover accelerates cultural evolution appears relatively modest (i.e., only the top left corner of Figure 2c), this is arguably the more realistic end of parameter space. This is because timesteps in the model are periods in which a single social learning event can occur. These are more likely to correspond to days or weeks rather than generations and, as such, population turnover per timestep in most populations will be very low (*m* << 0.1).

It is important to note that while population turnover can accelerate the adoption of the efficient solution, it will also place a constraint on the proportion of individuals in the population who possess it. Specifically, we find that the fraction of efficient individuals at equilibrium depends on the rate of turnover, *m*, and the rate at which individuals learn from an efficient individual once encountered, *s*_*e*_. When *m* ≤ *s*_*e*_, the fraction of efficient individuals at equilibrium is equal to 1–*m*/*s*_*e*_, that is, it is inversely proportional to turnover and directly proportional to the rate at which individuals learn from an efficient individual, if encountered. Notice that this means that efficient individuals can form half or more of the population at equilibrium only when *m* < *s*_*e*_/2. When *m* > *s*_*e*_, however, the efficient solution goes extinct, and only naïve individuals remain (see Figure 2d). This is because when *m* > *s*_*e*_, naïve individuals are being added to the population faster than they are adopting the efficient solution, and the pool of experience in the population will consequently be eroded. Thus, population turnover is a double-edged sword; it can accelerate the adoption of the efficient solution but also place a constraint on the maximum proportion of a population that can possess that efficient solution at any one time (see Figure 2e).

## DISCUSSION

Existing models showing that migration between groups accelerates the pace of cultural evolution assume that migrants can bring adaptive cultural traits with them (Powell et al. 2009; Creanza et al. 2017; Dyble 2018). But what if immigrants or juvenile recruits do not possess locally adaptive cultural knowledge? Our theoretical results demonstrate that even when newcomers do not arrive with adaptive traits, population turnover can still accelerate the adoption of an efficient cultural trait if newcomers are more open to social learning than established group members. These results help us to understand the effect of population turnover on cultural evolution generally and provide two important insights. First, we demonstrate that a cultural trait will become dominant only if the rate of social learning among naïve individuals is more than twice the rate at which knowledgeable individuals are replaced by naïve ones (Figure 2d). Second, we demonstrate the trade-off that is introduced by population turnover whereby increased turnover may increase the speed of adoption of a trait but also limit the maximum proportion of a population able to hold a cultural trait at any one time (Figure 2e).

Our model applies both to population turnover through migration, and through demographic turnover (i.e., births and deaths). In fact, demographic turnover is likely to be the more important process in many species, with cultural evolution driven by behaviorally conservative older individuals being replaced by juveniles receptive to social learning. For example, in the classic example of sweet-potato washing among Japanese macaques (Kawai 1965), the spread of the trait through the population from a single innovator to being held by the majority of group members was driven by the adoption of the trait among juveniles; 5 years following the innovation of the behavior, only 2 of 11 adults but 15 of 19 juveniles had acquired the potato washing behavior (Hirata et al. 2008). Adults who failed to acquire potato washing during the initial phase of transmission did not acquire it later, exhibiting strong behavioral conservatism (Hirata et al. 2008). Similarly, a recent study of the evolution of song types among Savannah sparrows (Williams et al. 2022) shows key features of the process explored in our model; juvenile males acquired song types through social learning, preferentially copied certain types, and after the acquisition of a song in the first year of life they were behaviorally conservative, repeating their song throughout their adult life. It is important to note that in the case of demographic turnover, cultural transmission may be vertical (from parent to offspring) as well as horizontal (among peers) and that a greater emphasis on vertical transmission is likely to slow down the rate of cultural transmission (Cavalli-Sforza and Feldman 1981; Kirby and Tamariz 2022).

Our model considers a simple cultural trait with only two solutions (inefficient and efficient). However, most cultural traits are much more complicated than this, and the ability of individuals to identify and learn efficient solutions may become more challenging when they are faced with a larger range of possible solutions. Many extensions to the existing model could be made, and further questions considered. For example, what would happen if the cultural trait could take more than two discrete forms? What if the efficiency of the cultural trait were continuous? What if rates of behavioral conservatism continued to increase with age? What if there was within-population heterogeneity in rates of social learning and in behavioral conservatism?

Although our results demonstrate that increased turnover can increase rates of cultural evolution, we also show that high levels of turnover can slow the pace of cultural evolution because if mortality and fertility are very high, naïve recruits are unlikely to encounter experienced individuals to learn from. This represents a key constraint on cultural evolution in short-lived species. In line with this point, most examples of non-human animal culture come from long-lived animals such as cetaceans, apes, and corvids (Whiten et al. 1999; Rendell and Whitehead 2001; Rutz et al. 2018; Aplin 2019; Whiten 2021; Garland et al. 2022). For humans, we suggest that the depth of a cultural trait (i.e., the proportion of a population who possess it) is less important for cultural adaptation than the speed of its adoption because with cumulative cultural evolution what is likely to be important is not that a trait reaches fixation but that a sufficiently large proportion of the population possess that trait to allow for some incremental improvement to be made on it. This “cultural ratchetting” is the driver of complex human culture (Tennie et al. 2009; Migliano and Vinicius 2022).

## Supplementary Material

arae003_suppl_Supplementary_Material

arae003_suppl_Supplementary_Data_S1

arae003_suppl_Supplementary_Data_S2

## Data Availability

Model scripts are available in the electronic supplementary material.
