# Supplementary material for: Population turnover, behavioral conservatism, and rates of cultural evolution
Source: Behav Ecol. 2024 Jan 17;35(2):arae003. doi: 10.1093/beheco/arae003 (PMC10807982; doi:10.1093/beheco/arae003)
Supplement: arae003_suppl_Supplementary_Material [file arae003_suppl_supplementary_material.pdf]

Supplementary Material for

Population turnover, behavioural conservatism, and rates of cultural evolution

Mark Dyble and Alberto J. C. Micheletti

1 Equilibria

The system of differential equations presented in the paper (eq. (1-3)) can be reduced to a system of two differential equations because at any time $e(t) + i(t) + n(t) = 1$. Replacing $e(t)$ with $1 - i(t) - n(t)$, we obtain:

$$i'(t) = -\mu\, i(t) - m\, i(t) + n(t)\, i(t)\, s_i - (1-c)\, i(t)\, \big(1 - i(t) - n(t)\big)(s_e - s_i) \tag{S1}$$

$$n'(t) = m\, i(t) + m\, \big(1 - i(t) - n(t)\big) - n(t)\, \big(1 - i(t) - n(t)\big)\, s_{\mathrm{e}} - n(t)\, i(t)\, s_{\mathrm{i}} \tag{S2}$$

In our analysis, we assume that the population is initially composed only of inefficient individuals, that is $e(0) = 0$, $i(0) = 1$ and $n(0) = 0$, because these conditions best describe the cultural evolution phenomenon we wish to model (see Methods section). However, for completeness, here we start by considering whether some initial conditions may be equilibria. If all individuals in the population are initially naïve, that is $e(0) = 0$, $i(0) = 0$ and $n(0) = 1$, it can be easily shown that, independently of the values of the parameters, $i'(0) = 0$ and $n'(0) = 0$. The system is at equilibrium and, from logical considerations, it is clear that this equilibrium is stable. The system does not change, because naïve individuals cannot learn the inefficient or efficient solutions independently (we assume no n-to-i or n-to-e innovation, see Methods section), but they can only learn them by copying inefficient or efficient individuals – and there are no models available when $n(0) = 1$. In all other cases, it can be easily shown that $i'(0)$ and $n'(0)$ do not simplify to zero for all parameter values, though they might for specific combinations of parameter values. We explore the possible equilibria when $n(0) \neq 1$ below.

The system is at equilibrium when $i'(t) = n'(t) = 0$. We indicate the values for the compartments at equilibrium by i^* and n^* (and $e^* = 1 - i^* - n^*$). Equations (S1-2) become:

$$-\mu\, i^* - m\, i^* + n^*\, i^*\, s_i - (1-c)\, i^*\, (1 - i^* - n^*)(s_e - s_i) = 0 \tag{S3}$$

$$m\, i^* + m\, (1 - i^* - n^*) - n(t)\, (1 - i^* - n^*)\, s_{\mathrm{e}} - n^*\, i^*\, s_{\mathrm{i}} = 0 \tag{S4}$$

We solve this system of two equations to obtain i^* and n^*. Equation (S3) is solved for $i^* = 0$. We substitute this value in equation (S4) and solve it, obtaining two solutions $n^* = 1$ and $n^* = m/s_e$. Considered that $0 \leq b^*, r^*, g^*, m, s_r \leq 1$, the solutions to the system of equations are:

$$e^* = 0 \qquad i^* = 0 \qquad n^* = 1 \tag{S5}$$

$$e^* = 1 - m/s_\mathrm{r} \qquad i^* = 0 \qquad n^* = m/s_\mathrm{e} \qquad \text{when } m \leq s_\mathrm{e} \tag{S6}$$

To determine if and when these equilibria are stable, we identify the conditions under which both eigenvalues of the Jacobian matrix for the model evaluated at these points are negative.

The Jacobian matrix is given by:

$$J = \begin{pmatrix} \frac{\delta i'(t)}{\delta i(t)} & \frac{\delta i'(t)}{\delta n(t)} \\ \frac{\delta n'(t)}{\delta i(t)} & \frac{\delta n'(t)}{\delta n(t)} \end{pmatrix} =$$

$$\begin{pmatrix} -m - \mu + s_i n(t) + (1-c)(s_i - s_e)(2i(t) + n(t) - 1) & (c(s_i - s_e) + s_e)i(t) \\ -(s_i - s_e)n(t) & -m - s_\mathrm{e} - (s_i - s_e)i(t) + 2s_e n(t) \end{pmatrix} \tag{S7}$$

In the case where $i^* = 0$ and $n^* = 1$, the eigenvalues of the Jacobian matrix are $-m + s_\mathrm{e}$ and $-m + s_\mathrm{b} - \mu$. It can be easily shown that both are negative when $m > s_e$. In the case where $i^* = 0$ and $n^* = m/s_e$, the eigenvalues of the Jacobian matrix are $m - s_\mathrm{e}$ and $\left((s_i - s_e)(c(m - s_e) + s_e)\right)/s_\mathrm{e} - \mu$. It can be easily shown that both are negative whenever $\mu \neq 0$ and $c \neq 1$ (see also *Wolfram Mathematica* electronic supplementary material).

Therefore, the stable equilibria of the system are:

$$e^* = 0 \qquad i^* = 0 \qquad n^* = 1 \qquad \text{when } m > s_\mathrm{e} \tag{S5}$$

$$e^* = 1 - m/s_\mathrm{e} \qquad i^* = 0 \qquad n^* = m/s_\mathrm{e} \qquad \text{when } m \leq s_\mathrm{e}, \mu \neq 0, c \neq 1 \tag{S6}$$

This result is illustrated in figure 2e. Notice that these results hold for all initial conditions except $n(0) = 1$.

2 Effect of increasing turnover on the time when the efficient solution dominates

Our goal is to identify areas of the parameter space in which increasing population turnover, m, results in the efficient solution dominating the population ($e(t_h) = 1/2$) faster. Specifically, we want to identify the values of conservatism, c, and turnover, m, for which t_h does not change with m. Since our system cannot be solved analytically, we did this numerically in *Wolfram Mathematica* (see the *Mathematica* notebook included as an electronic supplementary material). Using values of t_h obtained varying c and m, we produced an interpolating function $thinterpolation(m, c)$. We then derived this with respect to m and set it to zero to obtain the set of values of c and m for which t_h does not vary with m (the black line in figure 2c). Figure S1 shows values of t_h as a function of c and m, for nine different values of s_e.

See the *Wolfram Mathematica* notebook (.nb format), included as an electronic supplementary material, for the code used to produce the figures in the paper and in the Supplementary Materials.

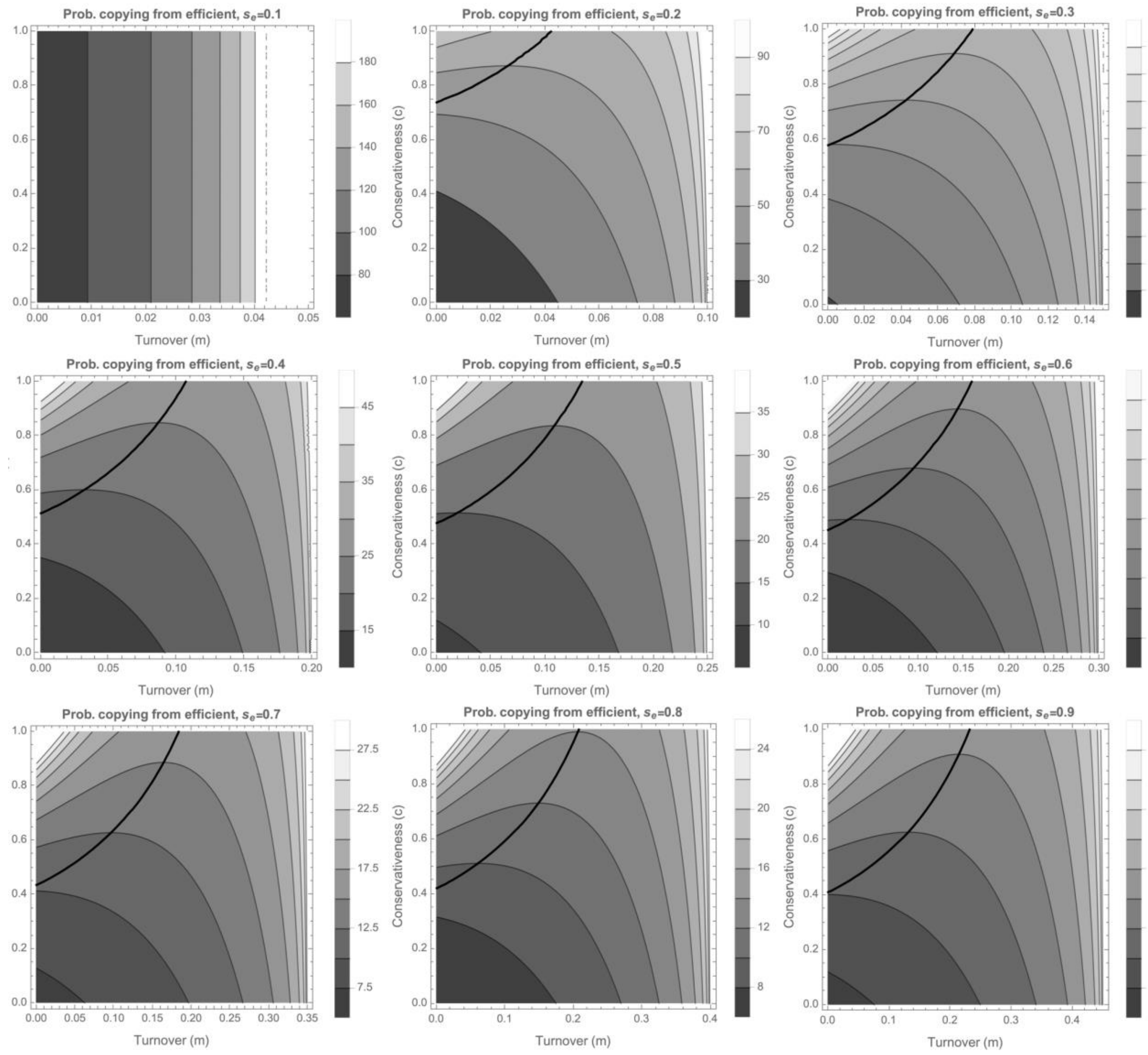

Figure S1 – Time at $e(t_h)$ = ½ (t_h, dark shades for low values, light shades for high values) as a function of population turnover (m) and behavioural conservatism (c), for different values of rate at which efficient individuals are copied (s_e). Above the black line, t_h decreases with increasing m, whereas below the black line t_h increases with increasing m. Parameter values μ = 0.01, s_i = 0.10, e_0=0, i_0=1, n_0=0.
