# Supplementary material for: Population turnover, behavioral conservatism, and rates of cultural evolution
Source: Behav Ecol. 2024 Jan 17;35(2):arae003. doi: 10.1093/beheco/arae003 (PMC10807982; doi:10.1093/beheco/arae003)
Supplement: arae003_suppl_Supplementary_Data_S2 [file arae003_suppl_supplementary_data_s2.pdf]

Population turnover model: results & figures

Wolfram Mathematica notebook (.nb) electronic supplementary material for “Population turnover, behavioural conservatism, and rates of cultural evolution”

by Mark Dyble & Alberto J. C. Micheletti

Notation

- $e(t)$ fraction of efficient individuals at time t
- $i(t)$ fraction of inefficient individuals at time t
- $n(t)$ fraction of naive individuals at time t
- e^*, i^*, n^* values at equilibrium
- μ mutation ($i \longrightarrow e$)
- m population turnover
- c behavioural conservatism
- si rate of learning from an inefficient individual once encountered
- se rate of learning from an efficient individual once encountered
- th time at which $e[th] = 1/2$

Parameter ranges and conditions

```
In[ ]:= rangesandconditions = {0 ≤ m ≤ 1, 0 ≤ c ≤ 1, 0 ≤ μ ≤ 1, 0 ≤ se ≤ 1, 0 ≤ si ≤ 1, se > si};
```

Differential Equations

The three differential equations

```
In[ ]:= ee = e'[t] == μ i[t] - m e[t] + (1 - c) (se - si) i[t] × e[t] + se n[t] × e[t];
In[ ]:= ei = i'[t] == -μ i[t] - m i[t] - (1 - c) (se - si) i[t] × e[t] + si n[t] × i[t];
In[ ]:= en = n'[t] == m (i[t] + e[t]) - si n[t] × i[t] - se n[t] × e[t];
```

Additional condition: fractions of efficient (e), inefficient (i) and naive (n) need to sum to 1 at any time

```
In[ ]:= ec = e[t] + i[t] + n[t] == 1;
```

Because of this condition, this system of equations can be reduced to a system of only two equations

```
In[•]:= substitution = {e[t] → (1 - i[t] - n[t])}
Out[•]=
{e[t] → 1 - i[t] - n[t]}
```

```
In[•]:= e2i = ei /. substitution
Out[•]=
i′[t] == -m i[t] - μ i[t] - (1 - c) (se - si) i[t] (1 - i[t] - n[t]) + si i[t] × n[t]
```

```
In[•]:= e2n = en /. substitution
Out[•]=
n′[t] == m (1 - n[t]) - si i[t] × n[t] - se (1 - i[t] - n[t]) n[t]
```

Equilibria: e^*, i^*, n^*

Finding the equilibria

Is the starting condition an equilibrium?

At $t = 0$, the differential equations are

```
In[•]:= e2i0 = e2i /. t → 0
Out[•]=
i′[0] == -m i[0] - μ i[0] - (1 - c) (se - si) i[0] (1 - i[0] - n[0]) + si i[0] × n[0]
```

```
In[•]:= e2n0 = e2n /. t → 0
Out[•]=
n′[0] == m (1 - n[0]) - si i[0] × n[0] - se (1 - i[0] - n[0]) n[0]
```

First, let us consider a case in which all individuals in the group are initially naive, that is $n[0] = 1$ and $i[0] = 0$. We obtain:

```
In[•]:= e2i0 /. {n[0] → 1, i[0] → 0}
Out[•]=
i′[0] == 0
```

```
In[•]:= e2n0 /. {n[0] → 1, i[0] → 0}
Out[•]=
n′[0] == 0
```

The system is at equilibrium and, from logical considerations, it is clear that this equilibrium is stable. The system does not change, because naive individuals cannot learn the inefficient or efficient solutions independently (we assume no n-to-i or n-to-e innovation, see Methods section), but they can only learn them by copying inefficient or efficient individuals — and there are no models available when $n[0] = 1$.

Second, let us consider a case in which initially individuals are either all efficient or a mix of efficient and naive individuals ($0 < e[0] \leq 1$, $0 \leq n[0] < 1$, and $i[0] = 0$). We obtain:

```
In[•]:= e2i0 /. {i[0] → 0}
Out[•]=
i′[0] == 0
```

In[ ]:=
```
e2n0 /. {i[0] → 0}
```
Out[ ]=
```
n′[0] == m (1 - n[0]) - se (1 - n[0]) n[0]
```

The system is not evidently at equilibrium (though it might be, depending on parameter values. We explore this in the next section)

Third, we consider a case in which $i[0] \neq 1$ and $n[0] \neq 1$. The differential equations at $t = 0$ are:

In[ ]:=
```
e2i0 // FullSimplify
```
Out[ ]=
```
i′[0] == i[0] (-m - μ + si n[0] - (-1 + c) (se - si) (-1 + i[0] + n[0]))
```

In[ ]:=
```
e2n0 // FullSimplify
```
Out[ ]=
```
m (-1 + n[0]) + si i[0] × n[0] + n′[0] == se n[0] (-1 + i[0] + n[0])
```

The system is not evidently at equilibrium (though it might be, depending on parameter values. We explore this in the next section)

The only case in which the system is evidently at equilibrium at the start is when $n[0] = 1$. We explore the possible equilibria when $n[0] \neq 1$ below.

Equilibria when $n[0] \neq 1$

At equilibrium $i'[t] = n'[t] = 0$.

$i'[t] = 0$ is solved for $i^* = 0$. As shown in the Supporting Information:

In[ ]:=
```
Block[{istar = 0},
 Reduce[{(e2i[[2]] /. {i[t] → istar, n[t] → nstar}) == 0,
   (e2n[[2]] /. {i[t] → istar, n[t] → nstar}) == 0}, {estar, nstar}]]
```
Out[ ]=
```
(se == 0 && m == 0) || (se ≠ 0 && nstar == m/se) || nstar == 1
```

There are two equilibria

- $i^* = 0$, $n^* = 1$ (and $e^* = 0$)
- $i^* = 0$, $n^* = m/se$ (and $e^* = 1 - m/se$), when $m \leq se$

Determining the stability of the equilibria

Equilibrium values i^* and n^* can only be asymptotically stable if both eigenvalues of the Jacobian matrix associated with the model are negative.

The Jacobian matrix is

In[ ]:=
```
jac = D[{e2i[[2]], e2n[[2]]}, {{i[t], n[t]}}];
jac // FullSimplify // MatrixForm
```
Out[ ]//MatrixForm=
```
( -m + (-1 + c) (se - si) - μ - 2 (-1 + c) (se - si) i[t] + (se - c se + c si) n[t]          (se - c se + c
                                  (se - si) n[t]                                         -m - se + (se - si) i
```

We now identify the conditions under which the two sets of equilibria identified in the previous section are stable.

$i^* = 0$, $n^* = 1$ (when $m \geq$ se)

The Jacobian matrix

```
In[]:= jac1 = jac /. {i[t] → 0, n[t] → 1};
       jac1 // MatrixForm
```

Out[]//MatrixForm=

$$\begin{pmatrix} -m + si - \mu & 0 \\ se - si & -m + se \end{pmatrix}$$

The eigenvalues are

```
In[]:= Eigenvalues[jac1] // FullSimplify
```

Out[]=

$$\{-m + se, -m + si - \mu\}$$

The condition for both eigenvalues to be negative is

```
In[]:= FullSimplify[Reduce[Eigenvalues[jac1] < 0],
        {0 ≤ m ≤ 1, 0 ≤ c ≤ 1, 0 ≤ μ ≤ 1, 0 ≤ se ≤ 1, 0 ≤ si ≤ 1, se > si, m ≥ se}]
```

Out[]=

se < m

Check

```
In[]:= FullSimplify[Reduce[Eigenvalues[jac1] < 0],
        {0 ≤ m ≤ 1, 0 ≤ c ≤ 1, 0 ≤ μ ≤ 1, 0 ≤ se ≤ 1, 0 ≤ si ≤ 1, se > si, m > se}]
```

Out[]=

True

The equilibrium $i^* = 0$, $n^* = 1$ is stable whenever $m >$ se

$i^* = 0$, $n^* = m/$se (when $m <$ se)

The Jacobian matrix

```
In[]:= jac2 = jac /. {i[t] → 0, n[t] → m / se};
       jac2 // MatrixForm
```

Out[]//MatrixForm=

$$\begin{pmatrix} -m - (1-c)\left(1 - \frac{m}{se}\right)(se - si) + \frac{m\,si}{se} - \mu & 0 \\ m - \frac{m\,si}{se} & -\left(\left(1 - \frac{m}{se}\right) se\right) \end{pmatrix}$$

The eigenvalues are

```
In[]:= Eigenvalues[jac2] // FullSimplify
```

Out[]=

$$\left\{m - se, -se + \frac{c\,(-m + se)\,(se - si)}{se} + si - \mu\right\}$$

The condition for both eigenvalues to be negative is

```
In[]:= FullSimplify[Reduce[Eigenvalues[jac2] < 0],
        {0 ≤ m ≤ 1, 0 ≤ c ≤ 1, 0 ≤ μ ≤ 1, 0 ≤ se ≤ 1, 0 ≤ si ≤ 1, se > si, m < se}]
```

Out[]=

(si == 0 && c se < c m + se + μ) || (si > 0 && (c (m - se) + se) (se - si) + se μ > 0)

Excluding cases when $\mu = 0$ and $c = 1$

In[∘]:=
```
FullSimplify[Reduce[Eigenvalues[jac2] < 0],
 {0 ≤ m ≤ 1, 0 ≤ c < 1, 0 < μ ≤ 1, 0 ≤ se ≤ 1, 0 ≤ si ≤ 1, se > si, m < se}]
```
Out[∘]=
```
True
```

The equilibrium $i^* = 0$, $n^* = m/\text{se}$ is stable whenever $\mu \neq 0$ and $c \neq 1$.

Figure 2a — Example with no turnover

In[∘]:=
```
Block[{μ = 0.01, m = 0.00, c = 0.75, si = 0.1, se = 0.5, e0 = 0.00, i0 = 1, n0 = 0, tmax = 50},
 soln = NDSolveValue[
   {ee, ei, en, e[0] == e0, i[0] == i0, n[0] == n0}, {e[t], i[t], n[t]}, {t, 0, tmax}];
 figure2a000 = Plot[{0.5, soln[[1]], soln[[2]], soln[[3]]}, {t, 0, tmax}, AxesLabel → {"t"},
   PlotStyle → {{Lighter[Gray, 0.6]},
     {Black, Thick}, {Black, Thick, Dashed}, {Black, Thick, Dotted}},
   PlotRange → {{0, tmax}, {0, 1}}, AspectRatio → 1 / 3, ImageSize → 400, Frame → True]]
```
Out[∘]=

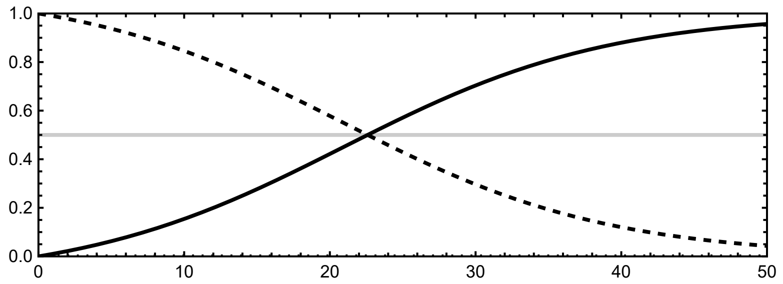

Value of *th*

In[∘]:=
```
Block[{μ = 0.01, m = 0.00, c = 0.75, si = 0.1, se = 0.5, e0 = 0.00, i0 = 1, n0 = 0, tmax = 50},
 soln = NDSolveValue[
   {ee, ei, en, e[0] == e0, i[0] == i0, n[0] == n0}, {e[t], i[t], n[t]}, {t, 0, tmax}];
 FindRoot[{soln[[1]] - 0.5}, {t, 1}][[1, 2]]]
```
Out[∘]=
```
22.5901
```

Figure 2b — Example with turnover

In[∘]:=
```
Block[{μ = 0.01, m = 0.10, c = 0.75, si = 0.1, se = 0.5, e0 = 0.00, i0 = 1, n0 = 0, tmax = 50},
 soln = NDSolveValue[
   {ee, ei, en, e[0] == e0, i[0] == i0, n[0] == n0}, {e[t], i[t], n[t]}, {t, 0, tmax}];
 figure2b010 = Plot[{0.5, soln[[1]], soln[[2]], soln[[3]]}, {t, 0, tmax}, AxesLabel → {"t"},
   PlotStyle → {{Lighter[Gray, 0.6]},
     {Black, Thick}, {Black, Thick, Dashed}, {Black, Thick, Dotted}},
   PlotRange → {{0, tmax}, {0, 1}}, AspectRatio → 1 / 3, ImageSize → 400, Frame → True]]
```

Out[∘]=

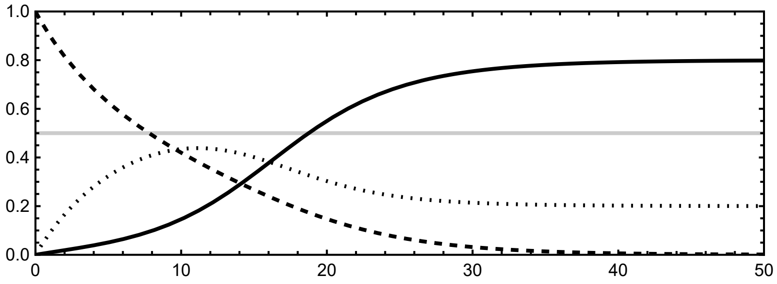

Value of *th*

In[∘]:=
```
Block[{μ = 0.01, m = 0.10, c = 0.75, si = 0.1, se = 0.5, e0 = 0.00, i0 = 1, n0 = 0, tmax = 50},
 soln = NDSolveValue[
   {ee, ei, en, e[0] == e0, i[0] == i0, n[0] == n0}, {e[t], i[t], n[t]}, {t, 0, tmax}];
 FindRoot[{soln[[1]] - 0.5}, {t, 1}][[1, 2]]]
```

Out[∘]=

```
18.7473
```

Figure 2e — Trade-off between depth and speed

The code to generate a plot with two vertical scales was adapted from https://reference.wolfram.-com/language/howto/GeneratePlotsWithTwoVerticalScales.html

In[∘]:=
```
TwoAxisPlot[{f_, g_}, {x_, x1_, x2_}] :=
 Module[{fgraph, ggraph, frange, grange, fticks, gticks},
  {fgraph} = MapIndexed[Plot[#, {x, x1, x2}, Axes → True,
      PlotStyle → {Black, Thick}, PlotRange → {{0, 0.2}, {0.5, 1}}] &, {f}];
  {ggraph} = MapIndexed[Plot[#, {x, x1, x2}, Axes → True,
      PlotStyle → {Black, Thick, DotDashed}, PlotRange → {{0, 0.2}, {10, 23}}] &, {g}];
  {frange, grange} = (PlotRange /. AbsoluteOptions[#, PlotRange])[[2]] & /@
    {fgraph, ggraph};
  fticks = N@FindDivisions[frange, 5];
  gticks = Quiet@Transpose@{fticks,
      ToString[NumberForm[#, 2], StandardForm] & /@ Rescale[fticks, frange, grange]};
  Show[fgraph,
   ggraph /. Graphics[graph_, s___] :→ Graphics[GeometricTransformation[graph,
       RescalingTransform[{{0, 1}, grange}, {{0, 1}, frange}]], s],
   AspectRatio → 1, Axes → False, Frame → True, FrameStyle →
    {{Darker[Gray, 0.2], Darker[Gray, 0.2]}, {Automatic, Automatic}},
   FrameTicks → {{fticks, gticks}, {Automatic, Automatic}}]]
```

In[ ]:=
```
Block[{μ = 0.01, ν = 0.00, (*m=0.1,*) c = 0.75,
  si = 0.1, se = 0.5, e0 = 0.00, i0 = 1, n0 = 0, tmax = 50},
 TwoAxisPlot[{1 - m / se, soln = NDSolveValue[
     {ee, ei, en, e[0] == e0, i[0] == i0, n[0] == n0}, {e[t], i[t], n[t]}, {t, 1, tmax}];
FindRoot[{soln⟦1⟧ - 0.5}, {t, 2}]⟦1, 2⟧}, {m, 0, se / 2}]]
```

Out[ ]=

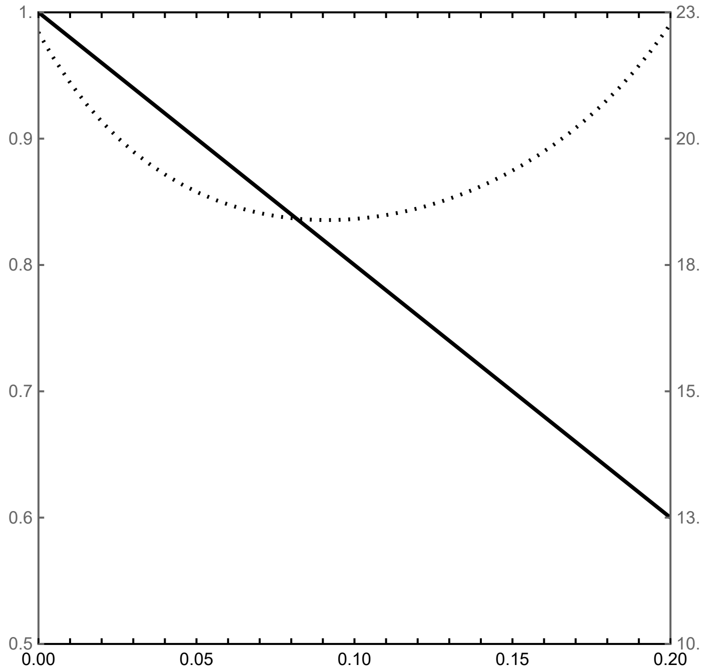

Figure 2c — t_h as a function of *m* & *c*

The function timehalf returns the value of *th* (that is the time at which *e*[*t*] = *i*[*t*] + *n*[*t*] = 1/2) for given values of *μ*, *m*, c, *si*, *se*, *e0* (*e*[0]), *i0* (*i*[0]), *n0* (*n*[0]). It does so by solving the system of differential equations numerically for values of t from 0 to a value tmax. The integration stops when the value *th* is reached.

In[ ]:=
```
timehalf[m0_, c0_, μ0_, si0_, se0_, e0_, i0_, n0_, tmax_] := Block[
{μ = μ0, m = m0, c = c0, si = si0, se = se0, t}, Reap[NDSolveValue[
      {ee, ei, en, e[0] == e0, i[0] == i0, n[0] == n0,
       WhenEvent[e[t] == i[t] + n[t], {Sow[t], "StopIntegration"}]},
      {e, i, n}, {t, 0, tmax}]] // Last // Last // Last]
```

We plot values of *th* varying *c* and *m* in a contour plot. Then, to obtain the line where *th* does not vary with *m* (the black line in the plot) we:

1) generate an interpolating function “thinterpolation” from values of *th,* varying *c* and *m,* using the native *Mathematica* function “Interpolation”.

2) take the partial derivative of thinterpolation[*m*,*c*] with respect to *m.*

3) Using “ContourPlot” we obtain the line where the derivative in 2) is equal to zero, i.e. the line where *th* does not vary with *m*

N.B. The following computation takes about **10 minutes** on a machine with Intel i7 and 16GB RAM.

In[ ]:=
```
Block[
{μ = 0.01, si = 0.1, se = 0.5, e0 = 0, i0 = 1, n0 = 0, tmax = 200, step = 0.001},

 (*controuplot of timehalf*)
 timehalfplot = ContourPlot[timehalf[m, c, μ, si, se, e0, i0, n0, tmax],
   {m, 0, se / 2}, {c, 0, 1}, PlotLegends → True, ImageSize → 300,
   ColorFunction → (Blend[{Darker[Gray, 0.5], White}, #] &),
   ColorFunctionScaling → True, PerformanceGoal → "Quality", MaxRecursion → 3];

 (*interpolating function*)
 thinterpolation =
  Interpolation[Flatten[Table[{{m, c}, timehalf[m, c, μ, si, se, e0, i0, n0, tmax]},
     {m, 0, 1 / 2, step}, {c, 0, 1, step}], 1], InterpolationOrder → 1];

 (* derivative of thfunction with respect to m*)
 pdm[m_, c_] := Evaluate[D[thinterpolation[m, c], m]] ;

 (*black line*)
 line = ContourPlot[pdm[m, c] == 0, {m, 0, 1 / 2},
   {c, 0, 1}, ContourStyle → {Black, Thick}, ImageSize → 300];

 figure2d = Show[{timehalfplot, line}]
]
```

Out[ ]=

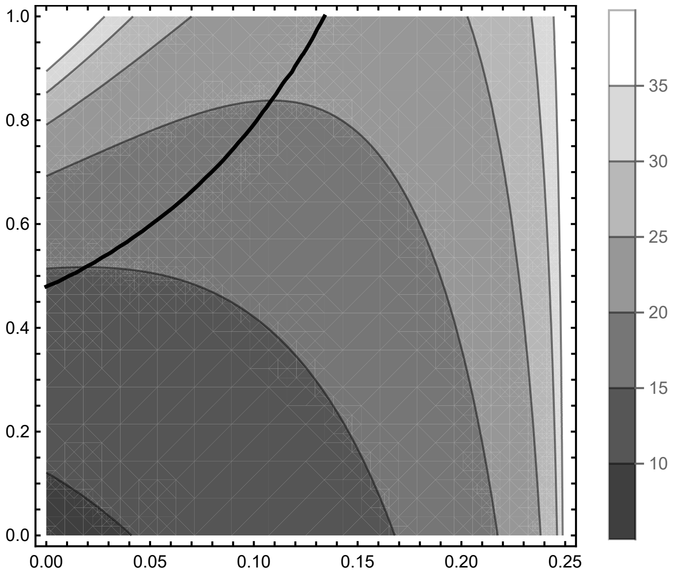

Figure 2d — equilibrium, r^*

In[∘]:=
```
Show[
 ContourPlot[1 - m/se, {m, 0, 1}, {se, 0, 1}, PlotLegends → True, ImageSize → 300,
  Contours → Range[0, 1, 0.1], ColorFunction → (Blend[{White, Black}, #] &),
  ColorFunctionScaling → False, PlotRange → All],

 Plot[{x, 2 x}, {x, 0, 1}, PlotRange → {{0, 1}, {0, 1}},
  PlotStyle → {{Thickness[0.007], Black}, {Thickness[0.007], Black}}]]
```

Out[∘]=

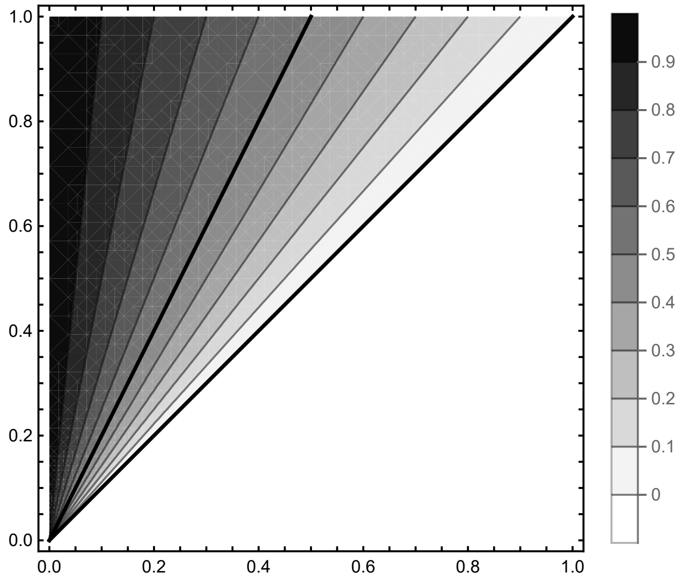

Figure S1 — t_h as a function of m and c, varying se

In[∘]:=
```
timehalf[m0_, c0_, μ0_, si0_, se0_, e0_, i0_, n0_, tmax_] := Block[
 {μ = μ0, m = m0, c = c0, si = si0, se = se0, t}, Reap[NDSolveValue[
      {ee, ei, en, e[0] == e0, i[0] == i0, n[0] == n0,
       WhenEvent[e[t] == i[t] + n[t], {Sow[t], "StopIntegration"}]},
      {e, i, n}, {t, 0, tmax}]] // Last // Last // Last]
```

N.B. The following computation takes about **6 hours** on a machine with Intel i7 and 16GB RAM.

```
(*Effect of turnover & conservativeness on timehalf*)
Block[
 {μ = 0.01, si = 0.1, e0 = 0, i0 = 1, n0 = 0, tmax = 200, step = 0.001},

 graphs = Table[

   (*controuplot of timehalf*)
   timehalfplot = ContourPlot[timehalf[m, c, μ, si, se, e0, i0, n0, tmax],
     {m, 0, se / 2}, {c, 0, 1}, PlotLegends → True, FrameLabel →
      {Style["Turnover (m)", Medium], Style["Conservativeness (c)", Medium]},
     ImageSize → 300, ColorFunction → (Blend[{Darker[Gray, 0.5], White}, #] &),
     ColorFunctionScaling → True, PerformanceGoal → "Quality",
     MaxRecursion → 3, PlotLabel →
      Style[StringForm["Rate of copying from efficient, se=``", se], Bold]];

   (*interpolating function*)
   thinterpolation =
    Interpolation[Flatten[Table[{{m, c}, timehalf[m, c, μ, si, se, e0, i0, n0, tmax]},
       {m, 0, 1 / 2, step}, {c, 0, 1, step}], 1], InterpolationOrder → 1];

   (* derivative of thfunction with respect to m*)
   pdm[m_, c_] := Evaluate[D[thinterpolation[m, c], m]] ;

   (*black line*)
   line =
    ContourPlot[pdm[m, c] == 0, {m, 0, 1 / 2}, {c, 0, 1}, ContourStyle → {Black, Thick},
     FrameLabel → {"Turnover (m)", "Conservatism (c)"}, ImageSize → 300];

   Show[{timehalfplot, line}],

   {se, 0.1, 0.1 + 0.1 * 8, 0.1}];
 graphs = Partition[graphs, 3];
 figures1 = Grid[graphs]
]
```

Out[•]=

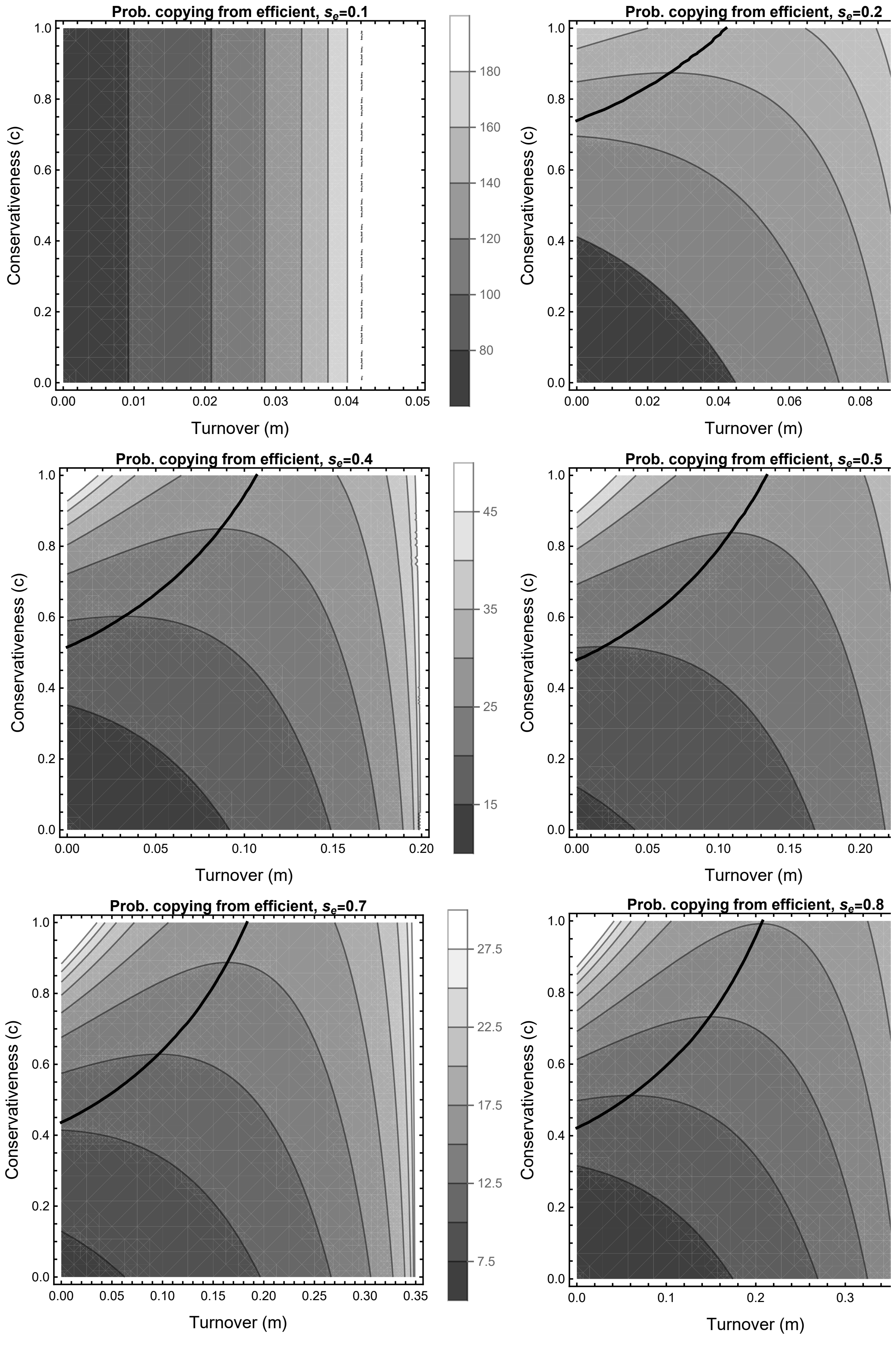
Prob. copying from efficient, s_e=0.1
Conservativeness (c)
Turnover (m)
180
160
140
120
100
80
Prob. copying from efficient, s_e=0.2
Conservativeness (c)
Turnover (m)
Prob. copying from efficient, s_e=0.4
Conservativeness (c)
Turnover (m)
45
35
25
15
Prob. copying from efficient, s_e=0.5
Conservativeness (c)
Turnover (m)
Prob. copying from efficient, s_e=0.7
Conservativeness (c)
Turnover (m)
27.5
22.5
17.5
12.5
7.5
Prob. copying from efficient, s_e=0.8
Conservativeness (c)
Turnover (m)
